# Epidemic Spreading on Preferred Degree Adaptive Networks

**DOI:** 10.1371/journal.pone.0048686

**Published:** 2012-11-26

**Authors:** Shivakumar Jolad, Wenjia Liu, B. Schmittmann, R. K. P. Zia

**Affiliations:** 1 Department of Physics, Virginia Tech, Blacksburg, Virginia, United States of America; 2 Indian Institute of Technology - Gandhinagar, Ahmedabad, Gujarat, India; 3 Department of Physics and Astronomy, Iowa State University, Ames, Iowa, United States of America; Universitat Rovira i Virgili, Spain

## Abstract

We study the standard SIS model of epidemic spreading on networks where individuals have a fluctuating number of connections around a preferred degree 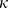. Using very simple rules for forming such preferred degree networks, we find some unusual statistical properties not found in familiar Erdös-Rényi or scale free networks. By letting 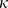 depend on the fraction of infected individuals, we model the behavioral changes in response to how the extent of the epidemic is perceived. In our models, the behavioral adaptations can be either ‘blind’ or ‘selective’ – depending on whether a node adapts by cutting or adding links to randomly chosen partners or selectively, based on the state of the partner. For a frozen preferred network, we find that the infection threshold follows the heterogeneous mean field result 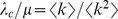 and the phase diagram matches the predictions of the annealed adjacency matrix (AAM) approach. With ‘blind’ adaptations, although the epidemic threshold remains unchanged, the infection level is substantially affected, depending on the details of the adaptation. The ‘selective’ adaptive SIS models are most interesting. Both the threshold and the level of infection changes, controlled not only by how the adaptations are implemented but also how often the nodes cut/add links (compared to the time scales of the epidemic spreading). A simple mean field theory is presented for the selective adaptations which capture the qualitative and some of the quantitative features of the infection phase diagram.

## Introduction

Concepts and tools from network science provide a powerful framework for the description of many physical, biological, and social systems, from the world wide web to neural architectures and from Facebook to power grids [1], [2]. In the initial years of the growth of network science, researchers focused on characterizing the network topology [1], [3], and then studying the time-dependent processes on complex static networks [4], [5]. Often the “dynamics *on* networks” was treated distinctly from the “dynamics *of* networks.” However many recent studies have focused on more realistic situations where dynamics *of* the network and dynamics *on* the network are coupled together, with a non-trivial feedback loop connecting them [6], [7]. In this work, we study the spreading of infectious diseases on a network of interpersonal connections where the adaptive behaviors of the affected population influence both the disease dynamics and the network topology.

The behavior of classic epidemic models such as susceptible-infected-susceptible (SIS) model and the susceptible-infected-recovered (SIR) model [8], [9] has been widely studied on regular lattices and on specific networks such as random, small world or scale-free networks [4], [10], [11] (see [12] for review). These studies assume that the disease spreads on a *static* network with characteristics which are *independent* of the nodes. However, in a dynamic social setting, people are likely to respond by social distancing or quarantine – changes in behavior that are perceived to reduce the likelihood of infection. Such behavioral adaptations will change the network topology and feed back into the dynamics of epidemic spreading. Recently, there has been growing interest to include such adaptive behavior in epidemic models. Given the wide range of human responses and their impact on the spread of the disease, modeling all these possibilities seems difficult and daunting. Thus, it is natural to consider simplified models with a few effective parameters. While such models cannot predict the epidemiological or social details quantitatively, they may be able to provide insight into qualitative and universal features of how adaptive behavior impacts the dynamics of epidemics. In this spirit, we introduce our models and study their properties.

Funk *et al* [13] classify the current literature on adaptive epidemic models based on the source of information (local or global) and the type of information (belief or prevalence) about the epidemic. Belief-based models emphasize individuals' awareness of a disease, and how they evaluate the associated dangers [14]–[17]. For example, some authors have modeled risk perception by decreasing the infection rate with the fraction of infected individuals in the local network of the node [18] and by introducing voluntary vaccinations [19], [20]. Prevalence-based models emphasize the objective assessment of the extent of epidemic spread and personal risk. Most of these studies have concentrated on coupling disease dynamics with network adaptations through rewiring of links [6], [7], [21]–[26] and studying the dynamics of S-I, S-S and I-I links. One might argue, however, that such rewiring models make a somewhat unrealistic assumption, namely, that individuals necessarily create a link with a healthy person after cutting a link with an infected one.

We address some of the limitations of prevalence-based epidemic studies by proposing a new type of network which contains a natural parameter, 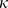, the ‘preferred degree.’ An individual (a node) with more/fewer contacts than 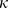 will tend to cut/add links. This parameter allows us to easily model *adaptive* behavior depending on the (perceived) level of threat from an epidemic. Let us point out several other advantages of this approach. Our network does not have unrealistically large degrees responsible for epidemics with vanishing thresholds [27]. Our model can easily be generalized to endow different nodes with different 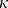
's, e.g., to account for the presence of extroverts and introverts [28], [29] in our society. Recent work has attempted to synthesize more realistic network such as those based on survey and census data [30], [31], and trajectories of mobile phone users [32]. Models based on realistic features of social network such as assortativity (homophily) [33] in social networks and range of interactions (like close and casual) have received considerable attention [34]. Our network model can be used to simulate features of these ‘realistic’ networks by making preferred degree distribution match the ‘true’ distribution and tuning the clustering coefficient by methods such as the one developed by Volz [35].

We highlight few major differences between our approach and the literature on prevalence and global information based adaptive networks. In the rewiring approach [6], [7], the total number of links in the population is *fixed for all time*, regardless of the level of the epidemic. By having a preferred-degree (which adapts to the state of the epidemic), the total number of contacts in the population is reduced when the disease spreads dramatically and returns to “normal” levels when the epidemic recedes. In this sense, our adaptive preferred degree plays a role analogous to the rewiring rate, in delaying the onset of an epidemic. Zanette and Risau-Gusmán [22] consider case where susceptible agents can decide to break links with their infected peers and links are permanently broken. In our approach, no link is permanently broken as the dynamics is kept active by infected nodes who can reconnect with any susceptible.

We begin by modeling the simplest case, where all nodes are characterized by a single 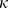, i.e., a homogeneous population. The network is dynamic, so that nodes can add or delete links, in an attempt to reach or maintain 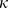. When a disease spreads on this network, the detailed dynamics of adding/cutting links changes in response to the epidemic. In the following, we propose a model reflecting *global prevalence-based* information, by letting 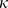 depend only on 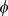, the fraction of infected individuals in the entire population. We model two typical human response: (a) If individuals are not aware who is infected and who is healthy (an ‘invisible’ disease, e.g., AIDS), they may cut (or add) links blindly in response to news of a raging epidemic. We will refer to this adaptive behavior as ‘blind response.’ (b) If the disease is ‘visible’ (e.g., the flu), an individual is more likely to be more discriminating when cutting or adding a contact – a response we naturally label as ‘selective.’ Here, the dynamics of network will depend on the state of the recipient node: Susceptible individuals will preferentially cut links with the infected and add links with other susceptibles. For the *blind* adaptations, we investigate three types of behavior: the *reckless* (where 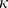 remains constant, then drops abruptly only when 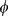 reaches some large value), the *typical* (where 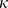 decreases linearly with increasing 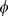, leveling off at some constant 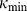), and the *nosophobic* (who cut ties precipitously as soon as 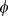 deviates from zero). We find that the epidemic threshold does not change, but the level of epidemic depends on the ‘degree of fear’ in the population. For the *selective* adaptations, we focus only on the reckless and typical types. Here, both the threshold and the level of infection change. We develop a mean field theory for local adaptations by writing equations for node and link dynamics. The predictions of this theory predict all the qualitative features of the simulations.

Our paper is organized as follows: In section I, we set the scene: presenting the formation of preferred degree networks and introducing an SIS dynamics on this network (initially with no adaptive features). We will summarize two theoretical approaches: a simple mean field theory (MF) and more sophisticated annealed adjacency matrix (AAM) method [36]. We also compare our results for the critical 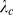 with predictions of heterogeneous mean field theory [37], [38]. In section II, we turn to study populations with adaptive response to a raging epidemic. In section III, we describe our main results for adaptive epidemic propagation. Section III.a deals with blind adaptations where a given nodes cannot “see” the disease states of the connected nodes.. The SIS phase diagram and degree distribution for these adaptive cases are much richer than those in non-adaptive networks. Much of the phase diagram is captured quite well by a simple mean field theory. In section III.b, we discuss the cases with selective adaptations. Simulation results are compared with a mean field theory, the details of which can be found in Appendix S1 (see supplementary information). We conclude, in the last section, with a discussion of our results and their implications for future research.

## Analysis

### I SIS on preferred degree networks

#### I.a Network formation

To explore the behavior of epidemics on dynamic networks, let us first present the foundation, i.e., a network with preferred degree(s). Following the lines introduced in [28], we briefly review how such a network is formed and evolves. Details of the statistical properties of such networks are also of interest, but will be presented in another publication [29]. For simplicity, we first consider a homogeneous population, i.e., a system with 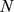 nodes (individuals) of identical behavior, evolving stochastically. In each time step, a random node 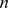 (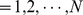) is selected and its degree, 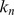, is noted. Then, an attempt to add (cut) a link is made, with probability 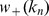 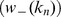 . Although an infinite variety of 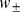
's is possible, we impose some general properties which mimic typical human behavior, e.g., 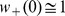 and 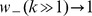, as well as the logical constraint 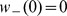. A simple choice, used in all our simulations, is 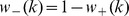, with
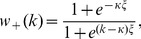
(1)recognizable as a Fermi-Dirac function. Here, 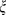 plays the role of ‘inflexibility’ (or ‘rigidity’) of the personality, so that a node (individual) with 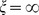 will always cut/add a link when it finds itself with more/fewer links than 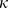. Indeed, apart from a brief digression in the next paragraph, the step function is used in all the simulations presented here. In the code, we choose 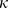 to be slightly larger than an integer, so that a node with 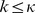 will attempt to add a link. Note also that, with 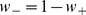, the network will always change, by the addition or deletion of a link. The partner node for this action is randomly chosen out of the eligible pool. Thus, the ‘recipient’ has no control over a link to it, whether created or destroyed. In a Monte Carlo step (MCS), 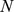 such attempts are made, so that there is one chance, on the average, for each node to add or cut a link.

**Figure 1:**
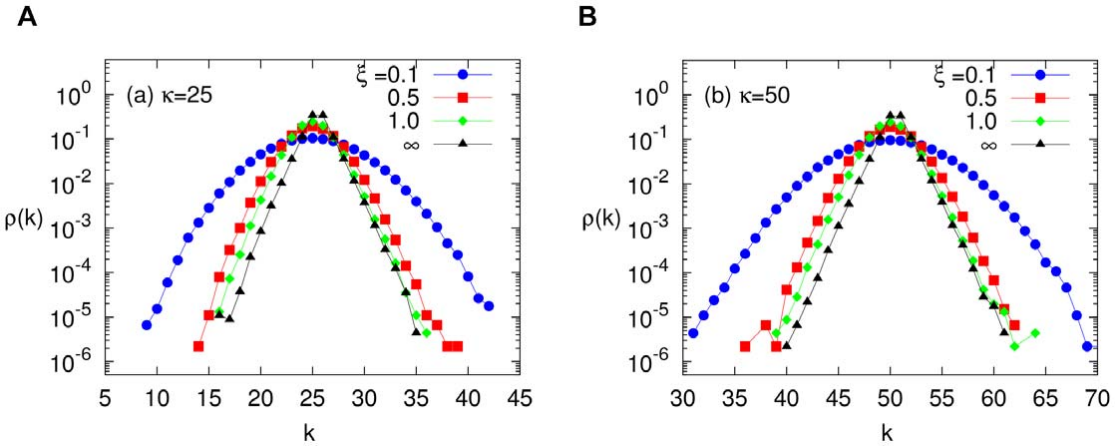
Degree distribution of preferred degree networks. Networks with 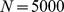 nodes and various inflexibility parameters 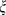 (see Eq. 1). Panels (a) and (b) corresponds to 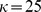 and 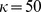 respectively. The 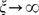 corresponds to the totally inflexible individuals and results in a Laplace distribution.

With a preferred degree, our network is clearly not scale-free. Also, unlike the case of a Erdös-Rényi network, the degree distribution in the steady state here, 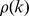, is *not Gaussian*. Though 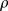 depends on the details of 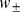, we discover a universal feature: exponential tails when 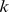 is far from 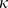. In Figure 1, we show typical simulation results for 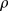 (with 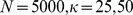). Indeed, for a group of completely rigid individuals (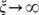), 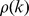 is a Laplace distribution (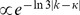). With a more flexible group (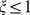), the maximum around 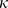 is rounded off, up to a width of 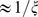, before crossing over to the same kind of exponential tails. This behavior is heuristically understood in the context of an approximate master equation, details of which can be found elsewhere [29], [39]. Our main focus in the remainder of this article will be the SIS dynamics associated with the nodes, evolving along with this changing network.

#### I.b SIS on static and dynamic preferred degree networks

Having presented the dynamics of a network with static nodes, we now endow the nodes with their own degrees of freedom. Following the standard SIS model [8], we assign a binary state variable, 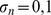, to node 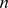, corresponding to that individual being susceptible (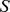) or infected (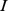). The system evolves by discrete attempts to update a randomly chosen node. If it is infected, then it recovers with rate 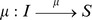. If it is susceptible, then the disease is transmitted with rate 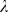 from each of its infected contacts 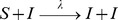 ( Here we set the time step equal to 1 making rates same as probabilities). We consider infection as a simultaneous event, so that an 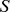 in contact with 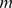 infected nodes will contract the disease with probability 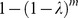 (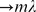 if 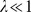). Again, a MCS is defined as 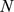 such attempts.

A good measure of the ‘level of the epidemic’ is the fraction of infected nodes: 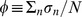. Clearly, a population with 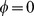 will not evolve, a state known as ‘absorbing.’ If the initial state has 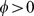, then the epidemic may die out (i.e., 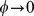) quickly or only over very long times, since there is a non-vanishing probability (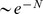) for a fluctuation to drop 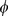 to 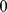. In the latter, known as an ‘active state,’ 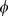 is typically positive, meaning that the epidemic is typically “ alive and well.” Whether the system becomes active or not will depend on network topology and the ratio 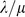. For simplicity, we fix 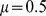 in all our simulations and use 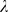 as a control variable. The goal is a phase diagram: Given 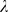 and a particular network, will the epidemic die or stay active? and where is its threshold: 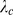
?

While a well-defined set of such questions can be formulated for infinite systems running for indefinite times, the task is less simple when confronted with simulations with finite systems and finite run times. In particular, since our systems will reach absorbing states in finite time, it is difficult to pin point the threshold, near which the typical 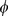 is vanishingly small. To overcome this difficulty, we introduced a trick into our simulations. To prevent our system from falling into the absorbing state, we do not allow the last 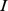 to recover. We refer to such a node as an ‘immortal’. We stress that we do not fix a single node as immortal, but simply prevent the last infected node from recovering. The advantage of this approach is clear: Our system never ceases to evolve, so that time averages in a steady state can be used to study ensemble averages (both denoted by 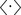). Of course, we should keep in mind that, in the ‘inactive state,’ 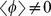 but 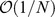. Further measurements can be implemented to characterize this state in more detail. For example, distributions of 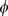 are expected to be exponential (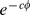) and how 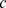 varies with 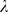 should be revealing.

We first studied *static* networks with a preferred degree, to provide a baseline for later investigations with co-evolving networks. For this study, we generated 50 network realizations using the scheme specified above (using 10K MCS for each run) and kept them quenched as we continued with the evolution of the nodes. After thermalization for 1000 MCS, we measure 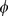 every 10MCS and then averaged over the 50 networks. The results for this (quenched) average 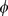, as a function of 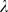, display a clear signal of the expected transition from inactive to active regimes of the epidemic. Away from 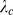, the fluctuations over a run are about 1%. The averages from the 50 realizations also do not differ by more than this amount. Not surprisingly, close to the transition, fluctuations are more substantial (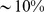). Exploring the critical region quantitatively is a worthwhile pursuit, but beyond the scope of this study.

**Figure 2:**
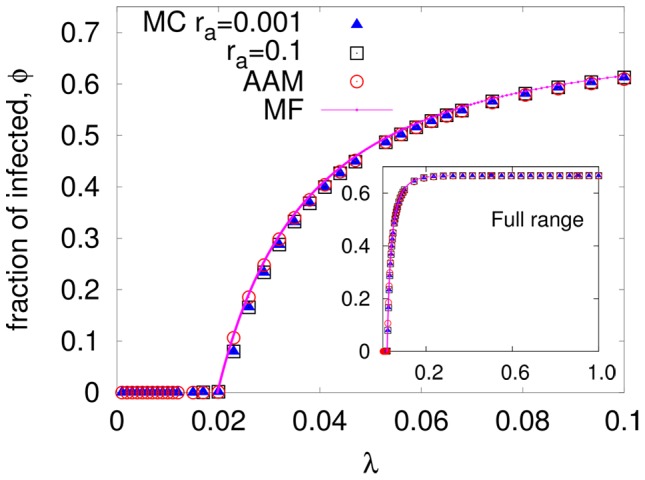
The SIS phase diagram for non-adaptive network. Fraction of infected population versus relative infection rate is plotted the vicinity of the transition point 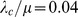 and compared with mean-field theories, for 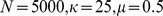, and two values of 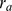. The numerically integrated AAM equations ( Eq 1. in [36]) are shown as open circles (red online), and results from the simple mean-field theory of Eq. 4 are plotted as solid lines (magenta online).

Next, we turn our attention to SIS on *dynamic* networks, where we must account for the fact that network and disease dynamics typically proceed at different time scales in society. Given that we are modeling the former as a *response* to a spreading epidemic, we will assume that network timescales are slower. In this spirit we choose the epidemic spreading to be 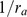 (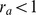) times faster than the network adaptations. That is, for every one MC step of the network, we perform 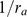 MCS of nodes. Mostly, we use 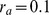. The SIS dynamics on a static network consists of letting 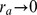. In practice, we performed runs with 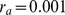 and found that 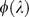 is not very sensitive to 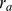 and that the 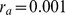 data are indistinguishable from those in static networks above. In Figure 2, we present results from runs with 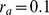 (open black squares) and 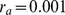 (solid blue triangles), leading us to the conclusion that, within our statistical errors, the time scales of network dynamics have little effect on an epidemic in a homogeneous population. We point to the readers that we present the results for time averaged data. Detailed investigations into the fluctuating dynamics is beyond the scope of the present work. For a recent work on instantaneous time description of network dynamics we refer the reader to [40]. In the next subsection, we will present theoretical perspectives of this system and how such phenomena can be understood.

#### I.c Simple mean field theory and the annealed adjacency matrix approach

To attack a statistical system theoretically, the first and simplest tool is a mean field (MF) approach. Since our interest is the long time behavior of 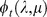, this first step consists of writing a simple equation for the evolution of 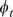. Following standard MF analysis, we write
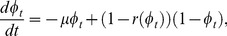
(2)where the first term models the 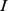
's recovering. In the second term, 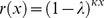 is the probability that an 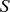 is *not* infected by any of its infected contacts. By setting the derivative to zero in Eq. 2, we find stationary solutions (fixed points): 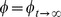. For small/large 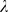, the stable 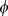 is zero/positive, corresponding to the inactive/active state. The transition is predicted to occur at
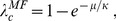
(3)which reduces, for 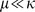, to an easily understandable result: 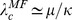. In the active state, 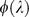 is given by the solution to 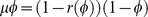. In other words, it is the inverse of the explicit 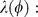


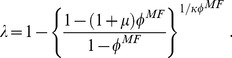
(4)The result is presented as the solid line (magenta on line) in Figure 2 and shows that, while slightly higher than the simulation results, it indeed captures the essentials of the epidemics. In the vicinity of criticality, the exponent in 
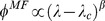
 takes the expected MF value 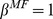.

In a dynamic or a quenched random network, this approach may seem too simplistic. In previous studies of SIS models on irregular, static networks, better approximations have been developed. Examples include the *heterogeneous* mean field (HMF) theory [37], [38] and the annealed adjacency matrix (AAM) approach [36]. The former takes into account a distribution of degrees, such as 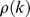 in our case, and provides the critical threshold at 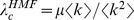, i.e., 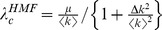. It has been widely applied, with considerable success, to study critical dynamics on various networks. For our study here, we present in Figure 1 the few cases of 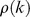 for the preferred degree networks used, showing that 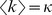 as expected and 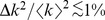. Hence, the simple MF prediction (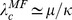) is quite adequate. Further, as our interest lies in the dominant behavior of the epidemic over the entire phase diagram, rather than details of the transition, there is no compelling need for using this complex method. As our network is dynamic, the AAM method may provide better predictions. Let us briefly summarize this approach [36] here. While the full dynamics involves a fluctuating adjacency matrix, in the AAM, the elements 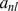 of the full fluctuating adjacency matrix are approximated by the probability that nodes n and l are connected. The infection probability of nodes are evolved through a discrete Markov equation (Eq. 1 in ref. [36]). Steady state values of infection probabilities are used to calculate 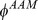. Applying this technique to our problem, we find that 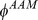 (red circles in Figure 2) follows 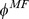 (magenta lines) quite closely at the transition region. As for 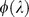 in higher 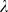
's, we show only the static network data and 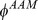 in the inset of Figure 2. As expected, the infected fraction simply saturates at 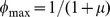. Clearly, the agreement between simulation results and all theoretical approaches is quite good. Thus, as a first step towards understanding epidemics on more complex, adaptive networks, we will rely on the simpler mean field theory.

### II Adaptive response to a raging epidemic

In the networks presented above, whether static or dynamic, the degree of each node is effectively fixed in time (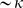 in our model). However, when an epidemic is present, individuals are likely to exhibit ‘social distancing’ behavior, by cutting ties or reducing the number of non-essential contacts (as documented in, e.g., [41], [42]). Apart from being an inherently natural response, cutting ties may also occur due to externally imposed public policies [41], [43]. When the state of the disease is not easily discernible (e.g., AIDS), one's response will be to sever links blindly. On the other hand, if the disease is ‘visible’ (e.g., the flu), one can be more selective, by cutting only contacts with the infected. Such adaptive behaviors can be easily accommodated in our model by letting 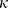 change, in response to the level of the infection. In this work, we will study the effects on the epidemic due to both ‘blind’ and ‘selective’ adaptations. In particular, we investigate infection levels, 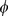, and degree distributions, 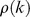, in the steady states.

#### II.a Models of response

**Figure 3:**
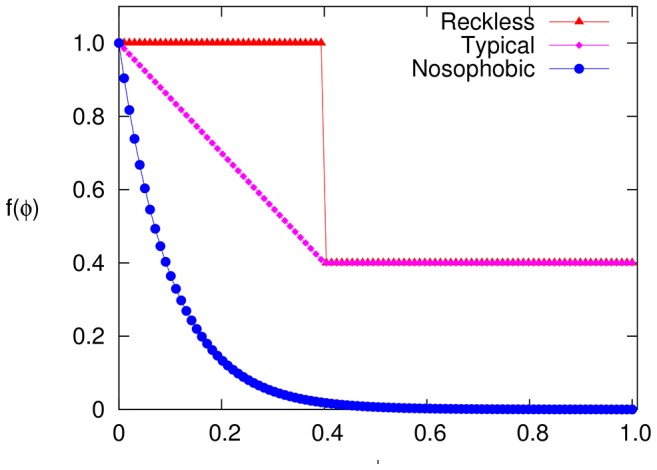
Adaptive fear factor. The “fear factor” 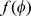 depending on the global infected fraction 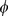 (see Eq. 5) associated with different behavioral patterns listed in section II.a.

To incorporate adaptive behavior, our first task is to specify how the population will lower the preferred degree, 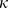, in response to a rising infection level. When an individual becomes aware of an epidemic, the response is likely a combination of rational/prudent behavior and irrational perceptions of the dangers. Though a typical population is diverse and heterogeneous, we begin with the simplest system: a homogeneous population with a unique response based on just one piece of information of the epidemic, namely, the global infection level 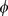. In other words, we let every node update with the same 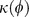. For convenience, 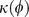 is introduced via a ‘fear factor’ 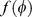:
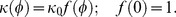
(5)Here, 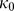 is just the preferred degree for an uninfected population, while 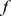 is a monotonically decreasing function, which serves to reduce the preferred degree. Of the infinitely many behavioral patterns that can be modeled, we consider only three kinds here (Figure 3):

- **Reckless** individuals are oblivious to a low level of epidemic present in the population. They keep the same 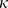 until the epidemic reaches a certain threshold: 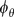. (We assume 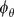 to be some fraction of 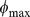.) At this point, they abruptly change their preferred degree to 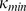. Keeping in mind that a typical person would maintain a minimal set of contacts (family, caretakers, etc.) even in the face of a raging epidemic, we simply choose 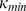 to be independent of 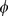 for all levels higher than 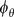. Explicitly, 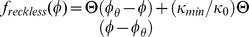, where 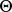 is the Heaviside step function. For simulations, we choose 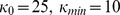, and 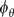 to be 60% of the maximum 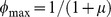. Since we fix 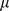 at 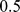, we use 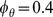.
- **Typical** individuals are likely to cut their contacts in a more measured fashion. For them, we choose a linearly decreasing 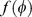. If this decrease is rapid enough, then these individuals' comfort level would reach the lower limit (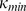) before the infection rate reaches its maximum level 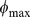. Again for simplicity, we let their 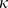 remain at 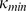 for all higher levels of infection. Explicitly, 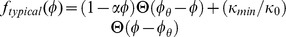, where the slope and the threshold are related by 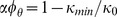. For this set of simulations, we chose the same parameters as above: 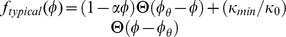.
- **Nosophobia** is an irrational fear of contracting diseases. To model such a population, we let 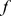 drop exponentially, as soon as the slightest infection is detected. These individuals would eventually avoid all personal contact. Explicitly, we have 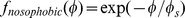. With 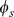 setting the severity of this phobia, we use 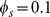 in our simulations.

Of course, any real population will have a mix of these behaviors, with perhaps time dependent compositions. Our hope is that studying these homogeneous cases separately will help us untangle the effect of different adaptive behavior on the epidemics. To summarize our model so far, when a node is chosen for updating its links, we measure its degree 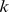 and take note of the overall infection level (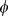). Then we add/cut a link if 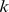 is less/greater than 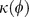. Choosing which link to add/cut and its affect on disease dynamics will be the focus of the next section.

## Results

### III Epidemic propagation in adaptive networks

#### III.a Blind adaptation

With an invisible disease, an individual does not know which of his/her contacts (or potential contacts) is infected. As a result, adapting to the news of say, a rising level of the epidemic, he/she simply cuts links to randomly chosen partners (as described in Section I) until a smaller 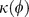 is reached. Similarly, if 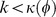, the new contact will be also chosen blindly. Setting aside the interesting question of how 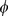 changes with time as a result of a changing network topology (in response to the feedback from 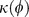), we focus on the steady states after the system settles down.

**Figure 4:**
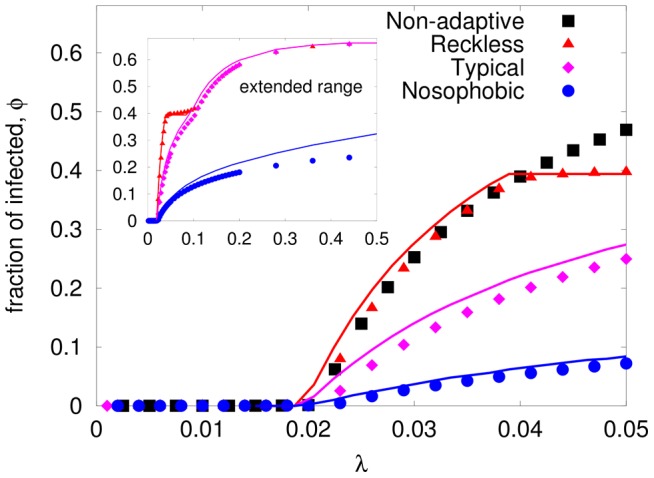
Non-adaptive and adaptive preferred degree SIS phase diagram. We have chosen 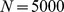, 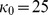 and 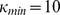 for all three adaptive models (See Figure 3). The solid lines represent the mean field solution to these models based on Eq. 6.

In Figure 4, we show the simulation results for 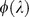 in these three cases (with mostly 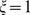, flexible individuals, for simplicity), as well as the case above: a non-adaptive network. We first observe that the epidemic thresholds are essentially unchanged by any of the adaptive strategies. This fact is understandable, since the threshold is defined by 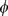 rising from zero and our transition is continuous. Thus, fear in the population has yet to take hold, and 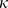 remains close to 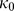. Beyond the threshold, the effects of the different fear factors are self-evident. The reckless follow the non-adaptive until 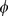 reaches 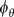 (chosen to be 0.4 here), and then abruptly adjust their response so that the infection remains more or less at this level. In the inset, we see that 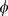 resumes its upward trend after 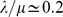, and reaches close to the maximal level 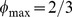 by 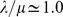. By contrast, the infection level in the typical case increases at a slower pace immediately after 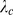. Around 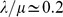, 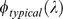 coincides with the reckless, since both networks are controlled by the same 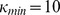. Finally, as expected, infections in a nosophobic population are strongly suppressed. Indeed, the critical properties near the transition may be altered. Since 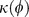 is effectively zero for 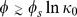 (i.e., 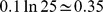 here), it is not surprising that the infection levels are far lower than the other two types.

More quantitatively, simple MF theory should provide an acceptable explanation for these results. From the analysis above, a 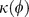 can be readily incorporated, so that 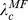 remains unchanged: 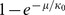 . Above this value, the only modification is the 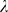-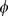 relationship, and Eqn. (4) now reads
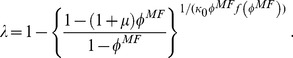
(6)Although the fear factor appears explicitly here, this expression is quite cumbersome. A simple way to regard the effects of adaptation is the following: To produce the same level of infection (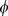), the infection rate (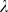) must be enhanced over the non-adaptive population. Quantitatively, 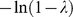 (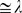, for small 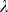 such as in our examples) must increase by a factor of 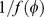. In this way, it is easy to see that the MF prediction of the critical exponent 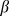 will remain unchanged, unless 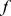 is appropriately non-analytic at 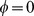 (i.e., 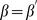 if 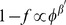 with 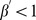). At the other extreme, the saturation levels are given by setting the left side of Eqn. (6) to unity. Unless the fear factor is so intense that 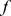 vanishes at a value of 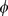 less than 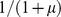, then, strictly speaking, these do not depend on the details of the adaptive strategy 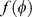. However, for the severely fearful such as the nosophobic, the infection essentially levels off at a 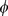 considerably lower than 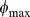.

Comparing with simulation data, we see that the MF predictions (Figure 4) tend to lie a little above simulation data, with the exception of few points near region associated with the abrupt drop in 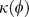 for the reckless population. We believe this effect may be the result of large fluctuations in the degree distribution. Individuals caught in this regime may cut ties drastically (at the news of 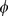 rising above 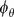), causing the infection to decline. But this good news would lead to the population reversing course just as abruptly, so that large fluctuations should continue. To test this conjecture, we now present degree distributions as an indication of how serious these fluctuations can be.

**Figure 5:**
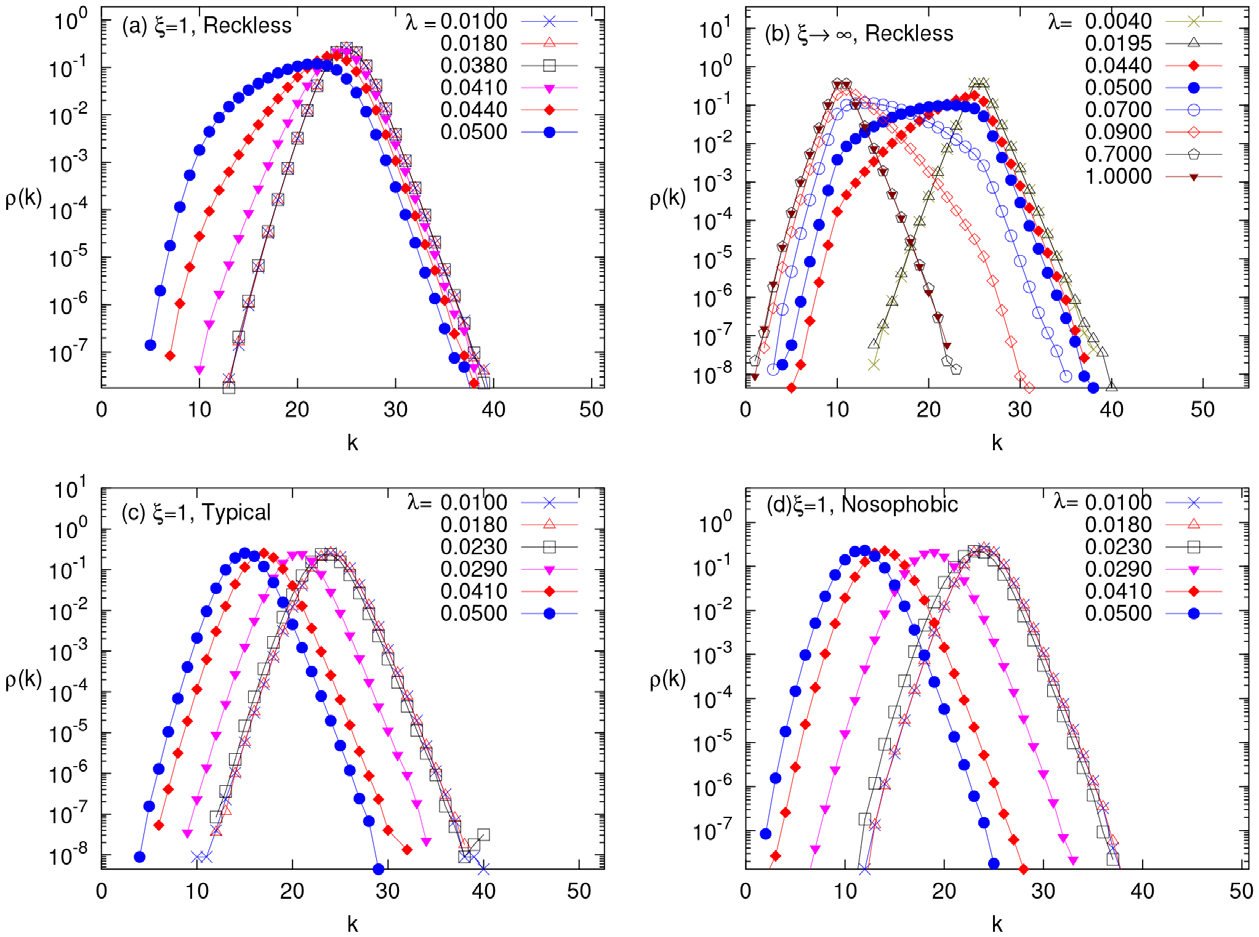
Steady state degree distribution of adaptive network. Degree distribution of (a) reckless with 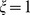 (see Eq. 1) (b) reckless and inflexible individuals (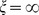), (c) Typical and (d) Nosophobic individuals (see Sec 3.A for details) with 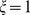. We have chosen 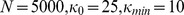 for all these cases. The infection rates 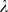 are chosen to illustrate transition behavior in degree distributions.

In the absence of infection, the degree distribution should be similar to those in Figure 1, around the preferred 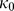. Far from the transition, the epidemic has settled in and, for both the typical and the reckless, the distribution should also be similar, but settling around 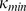 instead ( Figure 5 ). Not surprisingly, the picture is more complex for the nosophobic, especially for large 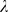, since the preferred degree is strongly dependent on the level of the infection and approach zero, which tends to isolating the nodes. Here, let us focus on the effects of the abrupt behavior of the reckless, the case that also displays the most interesting behavior (large fluctuations, Figure 5 a,b). For the other two types, we note the predictably mild changes in the degree distribution, as 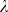 increases (Figure 5c,d). The overall *shape* of 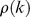 remaining essentially the same, but due to adaptations the center slowly shifts with 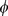 and 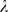.

For the reckless population, the conjectured behavior –dramatic swings when the infection level is near 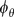, is well captured in the broadening of 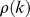. From the data shown in Figure 5a, we see that the distributions are, as expected, centered close to 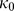 for 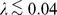 (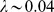 corresponding to the threshold 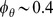). Thereafter, many individuals in the population begin to cut contacts. By 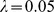, 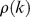 is quite distorted compared to the simple Laplace distribution. Specifically, we see that a sizable fraction of the population has cut their preferences down towards 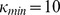. To display a complete range of infection rates, we chose to simulate with rigid individuals (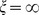) for simplicity (Figure 5b). Here, we see the complete crossover as 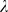 increases, from a distribution centered around 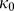 to one around 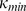. If we plot a reflected and appropriately shifted version of the 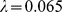 distribution (i.e., 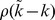 for an appropriate 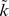), the result is essentially identical to the raw 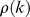 for 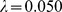. A similar collapse is observed for the cases with 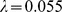 and 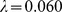, 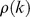. Thus, we may associate 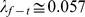 with a transition, from a population dominated by 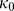 (i.e., non-adaptive behavior) to one controlled by 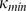 (i.e., typical). Since 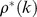 displays always a single peak, which shifted rapidly between 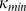 and 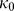, we would label this as a continuous transition.

#### III.c Selective adaptation

If the state of infected individuals is manifest (i.e., disease is ‘visible’), it is natural for individuals to be more selective in choosing their contacts. Such behavior might also be driven by policy interventions such as isolating the infected and/or closing public meeting grounds (e.g., schools) [41], [43]. In particular, how an individual adds/cuts links will now depend on the states of his/her contacts. We choose the following ‘think globally, act locally’ model which we believe is a reasonable representation of such adaptive behavior.

We initially set up a static preferred degree network with a preferred degree 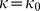. Infection is started in some fraction 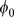 of the nodes and spreads according to the standard SIS dynamic rules described before. As in the blind adaptation case, the preferred degree 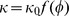 depends on the global infection level 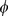. Unlike the previous method, when a node is chosen to update its links, the rules will depend on whether the node is susceptible or infected. Let us assume that an 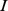 does not care about the state of the contacts and randomly adds/cuts links as before. However, an 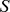 will behave more *selectively*, having a bias in favor of other 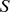
's after it decides to add or cut a link. To model this bias, we introduce a parameter, 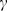, with which the favored choice is selected over the undesirable one. Letting subscripts denote the initiator-receptor pairs, 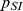 and 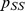 denote, respectively, the probability with which an 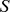 cuts a link to an 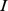 or an 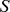. Obviously, we impose 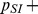 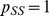. Similarly, let 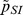 and 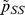 denote the probabilities it will *create*, respectively, a link to an 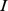 and an 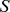 (with 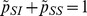). Explicitly, we choose the following.

- An 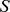 with degree 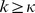 will *cut* a link from a randomly chosen 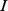 with probability
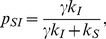
(7)or to a randomly chosen susceptible with probability 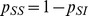. Here 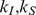 are the number of 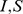 contacts it has. Now, it is clear that the larger 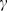 is, the more our 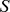 will choose to cut links to its infected contacts (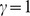 corresponds to non-preferential adaptation).
- Similarly, an 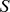 with degree 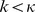 will *create* a link to a randomly chosen 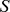 with probability
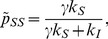
(8)or to a randomly chosen infected with probability 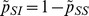. Again, we see a large 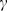 biases more towards adding links to other 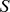
's.
- Since infected nodes do not have any incentive for selective adaptation, we make these nodes adapt blindly as follows:
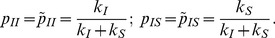
(9)

To allow for individuals to react at a different rate compared to that of recovery or infection, as in blind adaptation case, we update the links at a rate 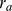 (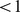) compared to the update of the state of the nodes.

With the rules described, we studied selective adaptations for reckless and typical cases (see section. II.a ) for moderate system sizes 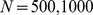. We found that system size satisfying 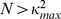 is sufficient to produce the ‘thermodynamic’ limit. While we note that steady state configuration depend only on the ratio 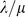, we alert the readers that our parameters 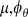 for selective adaptation are different from the blind adaptation case. We choose 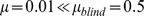, and the cut off infection level for 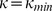 to be 60% of the maximum value 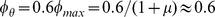.

**Figure 6:**
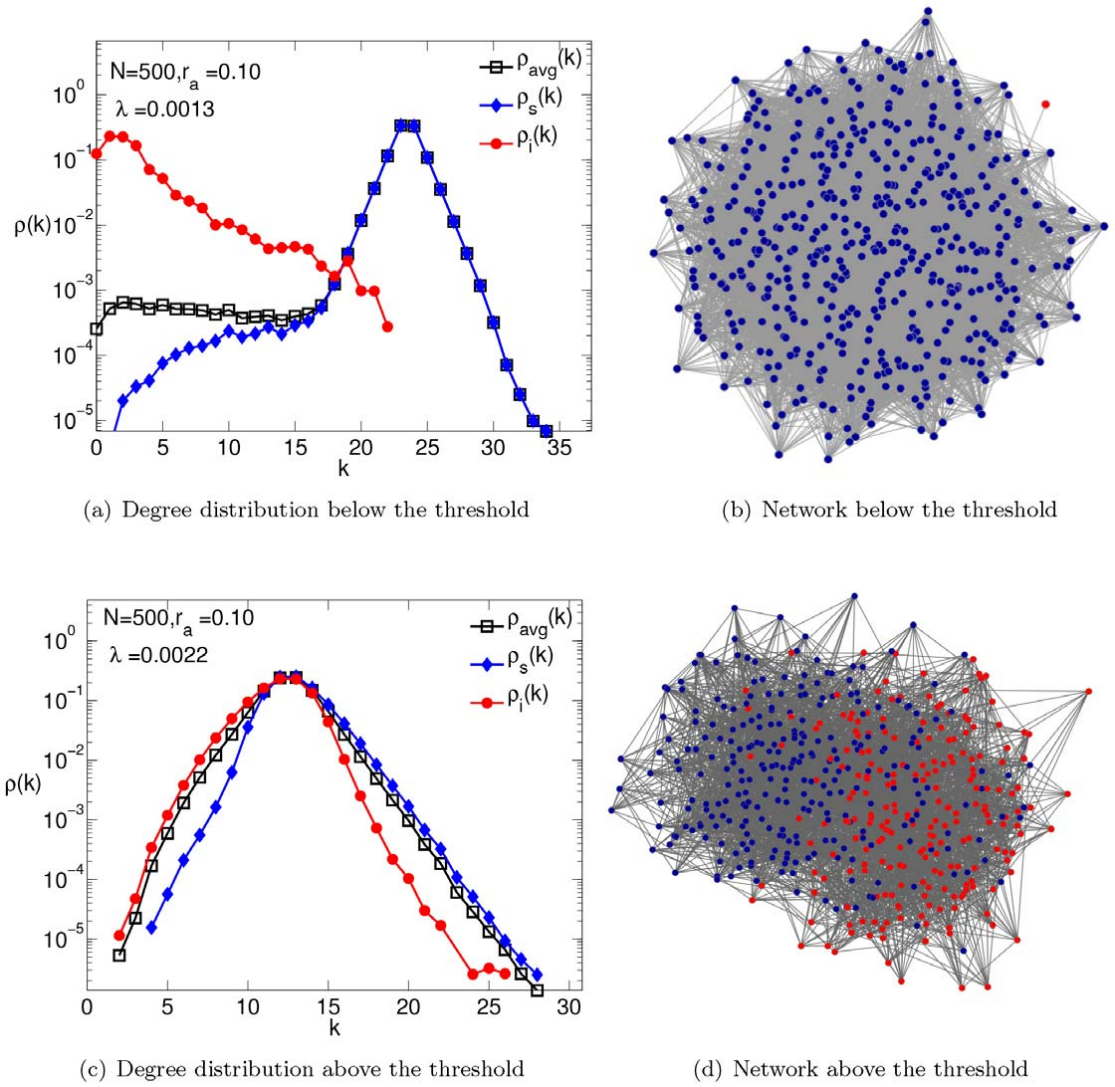
Degree distribution and network structures with typical local adaptations. Panels (a) and (b) show systems below the epidemic threshold, while (c) and (d) show systems above the threshold. The parameters chosen are 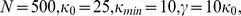 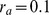, 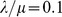.

In Figure 6a, we show the degree distribution of susceptibles, infected and total populations *below* the epidemic threshold for a typical behavioral adaptation case. Except for one immortal, the whole population is composed of susceptibles. The total degree distribution essentially reflects the susceptibles. However, the immortal can have different degrees during the course of SIS dynamics which will be reflected in the quenched distribution of infected. Figure 6b shows the network structure with the lone infected connected to the big cluster of susceptibles. In Figure 6c, we show the degree distribution of susceptibles, infected and total populations *above* the epidemic threshold with parameters 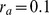 and 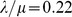. We see that all the degree distributions overlap. However the infected people are more strongly interconnected than with the susceptibles (see Figure 6d), which is indicated by non-zero modularity coefficient [44], [45] of Q = 0.2384.

**Figure 7:**
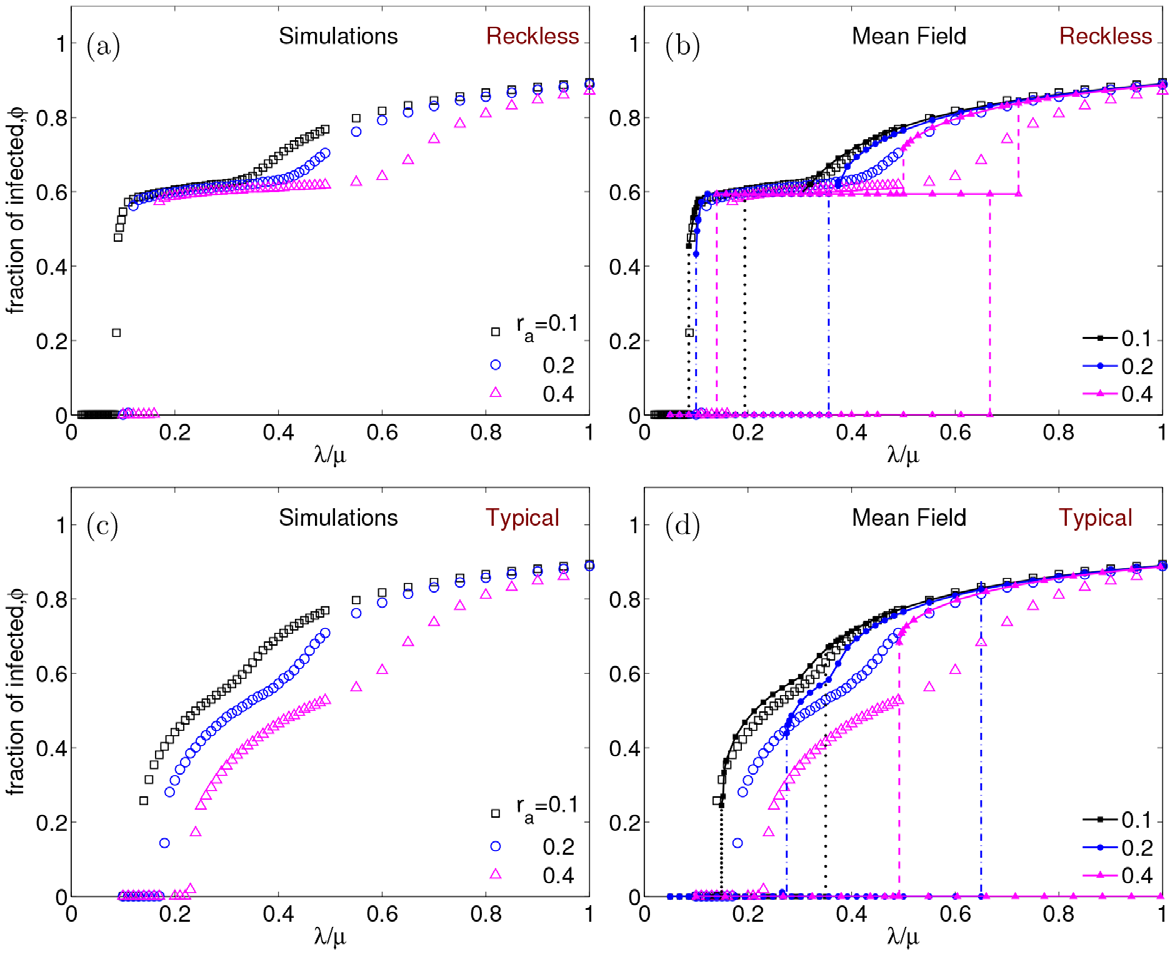
SIS phase diagram for selective adaptations. The fraction of infected population 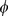, versus 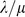 for different network adaptation rates, with parameters 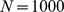, 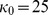, 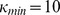, 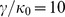. Panels (a) and (c) show the Monte-Carlo simulation results for reckless and typical behaviors (see Sec. II.a) respectively. In panels (b) and (d), the simulation results are compared to local mean field theory (described in Appendix S1) predictions. The black squares, blue circles and magenta triangles represents the network adaptation rates 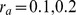 and 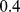 respectively. The corresponding mean fields results are plotted as lines with respective colors in (b) and (d). The dotted, dot-dash and dashed lines represent the bistable regions obtained from mean field solutions when initial infection fraction is varied from 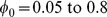 and initial links chosen from following the hysteresis curve.

In Figure 7a and c, we show the SIS phase diagram for reckless and typical adaptations obtained by Monte-Carlo simulations. In the figure, black squares, blue circles and magenta triangles correspond to relative network adaptation rates 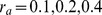 respectively. We observe that unlike the blind adaptation case, the epidemic threshold varies both with the network adaptation rate and behavioral response to different fear levels. The threshold increases with increasing 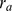 – an understandable feature, as faster responses by the 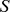
's should suppress the infection rates. In both cases, the transition from a healthy state to an active infectious state is considerably more rapid than in the blind adaption case. Indeed, for the reckless population with faster network response (larger 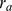), we observe a discontinuous transition (or a very steeply rising continuous one). In both cases, there is a second crossover, near 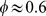, to a gently rising 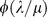 curve. These can be traced to our choice of 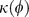, which contains a singularity (discontinuity or kink) at 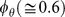.

Since the adaptation is in response to a ‘local’ environment of a susceptible individual, a more sophisticated mean field theory needs to be formulated. To distinguish this from the mean field approach above, we will refer to it as the ‘local mean field theory’ (LMFT). In particular, we introduce three more variables: 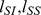, and 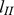, defined as the mean number of 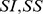 and 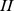 links per node, respectively. While the evolution equation for 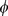 is just modified to be 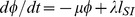, the equations for the 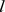
's are much more involved. Deferring to the Appendix S1 (see supplementary information ) the details of how these are formulated and studied, let us focus here on the results of the stationary solutions, Eqns. (A4, A11) of Appendix S1, and how they compare with simulation data. Illustrated in Figure 7b and d, the general conclusion is that there is reasonable qualitative agreement between LMFT and Monte Carlo results.

For the case with reckless adaptations, the response to infections is quite rich while the agreement is better than expected. In particular, LMFT predicts *three* stable fixed points: one associated with the inactive 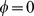, another associated with 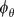, and the third, with a ‘normal’ endemic state. The presence of the second fixed point is probably the result of the discontinuity in our 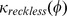. Moreover, for a moderate range of 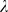, the LMFT displays bistability. Of course, in a stochastic simulation, one of these will be metastable with a discontinuous transition in 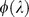. Such differences are common, much like bistability in a Landau theory of ferromagnetism below criticality *vs.* metastability/stability in a statistical system. Overall, we see that simulation data generally support the existence of *three* branches, in good agreement with LMFT. In more detail, we find that the nature of the first transition (threshold of the epidemic, from the inactive state to 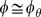) is well predicted by LMFT. Comparing the location of the discontinuous transition is, of course, very difficult. Nevertheless, simulations indicate these locations to lie within the LMFT limits of bistability. In any case, there is good reason to believe that the (bare) value of 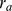 (from simulations) will be ‘renormalized’ by fluctuations, so that a better theory may converge towards the data. Turning to the second transition, at higher 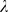, we see that it is associated with 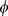 exceeding 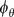, which in turn leads to a jump in 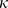 (from 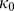 to 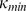). Thus, the network will become homogeneous again: With degree 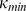, the theoretical 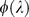 follows 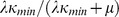. This prediction agrees with simulations, once 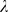 far exceeds the transition values. More intriguingly, LMFT predicts the nature of this transition to depend on 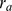. While it is a typical bifurcation for the lower 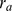
's, it a involves *tri-stability* region (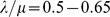), with all the three branches are stable for the 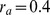 case. In the latter case, the LMFT displays oscillating time dependence in all the variables in the 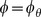 branch, pointing to the possibility of limit cycles and Hopf bifurcations. Perhaps just an artifact of the discontinuity in 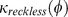, these fascinating aspects deserve further study. Comparisons with data are more ambiguous. For example, simulations favor gentle crossovers rather than discontinuities in 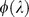 or 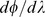. Remarkably, the location of these crossover are not too far from the transition predicted by the LMFT.

For the ‘typical’ adaptive behavior, we find two stable fixed points corresponding to the inactive or endemic states. Moreover, for a moderate range of 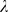, the LMFT displays bistability, i.e., it predicts a discontinuous transition. The agreement between LMFT and simulation results is arguably good for 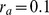, finding even the kink associated with 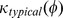 at 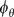. For larger 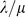, the branch of the LMFT bistable region and the data follows 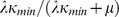 for all 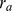
's. For the larger 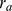
's, the theory continues to predict a discontinuous transition at the threshold, while the data show a steadily decreasing discontinuity. It is quite possible that these end on a multicritical point, beyond which the behavior is more typical of a ‘second order’ transition. Such subtle issues can only be clarified with a larger systematic simulation study. The reasons for the discrepancy between LMFT and simulations are unclear. We speculate that some of the approximations used were too crude, e.g., replacing the local degrees with the global averages (see Appendix S1 in supplementary information for details) and assuming degree distributions to adopt instantaneously to the steady state adaptive preferred degree (with a time dependent 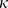). These are issues worthy of further investigation. Clearly, there is considerable room for improvement as many questions remain to be explored before we arrive at a satisfactory theory.

## Conclusions

The study of dynamical processes on networks has been very active for several decades. Most investigations have focused on either a dynamic set of nodes on a static network (e.g., spins on a lattice or epidemics in a population with fixed connections) or a dynamic network with static nodes (e.g., small world networks, scale free networks). Only recently have researchers focused their attention on dynamics of co-evolving networks where both nodes and links are dynamic, with particular attention to opinion dynamics and epidemic spreading. Here we consider the classic SIS model of epidemic spreading, on a network that adapts to the level of the infection. Introducing a new class of networks in which individuals (nodes) favor a certain number of contacts (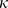, the preferred degree), we model various types of adaptive behavior by letting 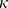 depend on the level of the epidemic, through 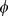, the infected fraction of the population. For such networks, we typically find degree distributions that are neither Gaussian nor scale-free. Instead, the universal feature appears to be exponential tails when the degree is far from 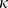.

Using Monte-Carlo methods, we simulated populations in which healthy individuals may become ill by being in contact with a fluctuating set of infected nodes, while diseased persons recover spontaneously with some rate. We considered three types of adaptive behavior representing the degree of fear in the public, which were modeled by different adaptive preferred degree as a function of global infection level. Further, these network adaptations can be *blind*, i.e., a central node does not know the disease state of its contacts, or *selective* where the disease state of the neighbors is known and the central node responds by selectively cutting or creating links. For the blind adaptations we find that the epidemic threshold does not change with the degree of fear, however the level of epidemic in the active phase decreases with increasing fearful response. A good agreement with the simulation data can typically be found with a simple mean field theory. For the selective adaptations, much more interesting dynamics emerge. The epidemic threshold changes substantially with increasing rate of network adaptations (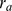). The epidemic transition is discontinuous, unlike the blind adaptation case which shows a continuous transition. The level of epidemic in the active phase changes with both the network adaptation rate and the degree of fear in the public. We have presented a local mean field theory with equations for both node and link dynamics for selective adaptations. For reckless and typical cases, it predicts bistable regions in which both, a healthy and an active infectious phase persist - a standard indicator of discontinuous transitions. There is qualitatively good agreement between mean field predictions and simulation data. Sources for the (quantitative) differences abound, from the crude level of approximations used to the subtle effects of fluctuations.

Within the scope of our study, many issues remain to be investigated and better understood. Clearly, our mean field treatment relied on significant approximations; how can this approach be improved? Do the observed discontinuous transitions share typical aspects of ‘first-order’ transitions, e.g., hysteresis and metastability? If so, does our system fall into the universality class of the standard SIS problem? Are there new exponents, associated with the network fluctuations and its dynamics? At a more detailed level, insights into much of the properties of the network (e.g., degree distributions, clustering, modularity, etc.), especially in the case with selective adaptations, would be very desirable.

Apart from the two types of adaptation we have presented, many extensions can be pursued. In a typical society, the population is inhomogeneous, so that an individual's perception of the infection level may not be the same as the overall 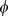. Letting the adaptive behavior depend on this perceived level, we consider variations in strategies by simply adding a white noise to 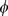. Our preliminary studies with ‘blind’ adaptations, not reported above, indicate that the effect of this type of noise on the epidemic appears to be minimal. Beyond our simple model, the most immediate generalization is to include spatial structures, both homogeneous and heterogeneous. For example, extroverts and introverts have very different preferred degrees. How does an epidemic develop across these different communities? There is a general belief that extroverts are more prone to contagious diseases. A further generalization would be to study epidemics on realistic networks with known degree distributions and clustering. Such networks can be synthesized by heterogeneous preferred degree networks with appropriate built through ‘small world’ algorithms. We postpone such work to a future publication. Naturally, the long term interest in such studies is to develop a good understanding so that reasonable public policies can be formulated in response to a real epidemic.

## Supporting Information

Appendix S1 **Local mean field theory for selective adaptation.**
(PDF)
