# Supplementary material for: Epidemic Spreading on Preferred Degree Adaptive Networks
Source: PLoS One. 2012 Nov 26;7(11):e48686. doi: 10.1371/journal.pone.0048686 (PMC3506630; doi:10.1371/journal.pone.0048686)
Supplement: Appendix S1 — Local mean field theory for selective adaptation. (PDF) [file pone.0048686.s001.pdf]

Appendix: Local mean field theory for selective adaptation

Here we discuss a formalism to understand the dynamics of global infection level and links for selective adaptation. We define the 'spin variable' $\sigma_n = \{0,1\}$ to denote the susceptibles (0) and infected (1) individuals of node n $(= 1, 2 \ldots, N)$. The fraction of infected individuals for any configuration is $\sum_n \sigma_n/N$. In the mean field approach, only its average is kept, so we simply replace $\frac{1}{N}\langle\sum_n \sigma_n\rangle \to \phi$.

Meanwhile, a network is uniquely specified by the adjacency matrix $a_{mn} = \{0,1\}$, representing the absence or presence of the (undirected) link between nodes m and n. The degree of node n is then given by $k_n = \Sigma_m a_{nm}$ so that the familiar degree distribution is given by $\rho(k) = \Sigma_n \delta(k_n - k)/N$. As we have two kinds of nodes, let us define two separate degree distributions, for the S's and the I's:

$$
\begin{aligned}
\rho_S(k) &= \frac{1}{N(1-\phi)} \sum_n (1-\sigma_n)\delta(k_n - k) \\
\rho_I(k) &= \frac{1}{N\phi} \sum_n \sigma_n \delta(k_n - k). \qquad \text{(A1)}
\end{aligned}
$$

Note that each is normalized, so that $\rho(k) = (1-\phi)\rho_S(k) + \phi\rho_I(k)$. Observe that the average of $\sum_{m,n} a_{mn}/2N$ is $\langle k\rangle/2$, which is, in a network with preferred degree, just $\kappa/2$.

Let l_{SI}, l_{SS}, l_{II} denote these averages *per node*, respectively:

$$
\begin{aligned}
l_{SI} &= \frac{1}{2N} \sum_{m,n} \left[(1-\sigma_m)a_{mn}\sigma_n + \sigma_m a_{mn}(1-\sigma_n)\right] \\
l_{SS} &= \frac{1}{2N} \sum_{m,n} \sigma_m a_{mn} \sigma_n \\
l_{II} &= \frac{1}{2N} \sum_{m,n} (1-\sigma_m) a_{mn} (1-\sigma_n). \qquad \text{(A2)}
\end{aligned}
$$

Thus, we should have

$$
l_{SI} + l_{SS} + l_{II} = \langle k\rangle/2. \qquad \text{(A3)}
$$

Turning to *dynamics*, the equation for ϕ is given, in the mean field approximation, by:

$$
\frac{d\phi}{dt} = -\mu\phi + \lambda l_{SI}. \qquad \text{(A4)}
$$

The dynamical equations for these links are more involved. For ease of understanding, we split the link equations into three parts, separating effects of node dynamics (infection, recovery) and network adaptations [1].

Node Dynamics: When the network topology is fixed, but the state of nodes are changing due to infection and recovery process, equations for the links can be written as:

$$
\begin{aligned}
\frac{dl_{SI}}{dt} &= \lambda\left[2\frac{l_{SS}l_{SI}}{1-\phi} - \frac{l_{SI}^2}{1-\phi} - l_{SI}\right] - \mu\left[l_{SI} + 2l_{II}\right] \qquad \text{(A5a)} \\
\frac{dl_{SS}}{dt} &= -2\lambda\frac{l_{SS}l_{SI}}{1-\phi} + \mu l_{IS} \qquad \text{(A5b)} \\
\frac{dl_{II}}{dt} &= \lambda\left[\frac{l_{SI}^2}{1-\phi} + l_{SI}\right] - 2\mu l_{II}. \qquad \text{(A5c)}
\end{aligned}
$$

Here we have used the standard moment closure approximation for triplets $l_{abc} = l_{ab}l_{bc}/l_b$, with a, b, c being S or I. [2,3]. The term $l_{SSI} \approx \frac{l_{SI}l_{SS}}{1-\phi}$ in Eq. A5a (b) corresponds increase (decrease) SI (SS) links

due to $[SSI]$ triplet. $l_{ISI} \approx \frac{l_{SI}^2}{1-\phi}$ corresponds to decrease in SI (increase in II) link. In both infection and recovery process, the total number of links is conserved.

Network Adaptations: But, our links are also being created and cut, at a rate r_a relative to the node dynamics, according to the rules of selective adaptation described in section III.C. Thus, we must add such terms to the dl/dt equations. Within the spirit of mean field theory, these are given by

$$\begin{aligned}
\frac{1}{r_a}\frac{dl_{SI}}{dt} &= \sum_k (1-\phi)\rho_S(k)\left[\Theta(\kappa-k)\tilde{p}_{SI} - \Theta(k-\kappa)p_{SI}\right] + \sum_k \phi\rho_I(k)\left[\Theta(\kappa-k)\tilde{p}_{IS} - \Theta(k-\kappa)p_{IS}\right] \\
\frac{1}{r_a}\frac{dl_{SS}}{dt} &= \sum_k (1-\phi)\rho_S(k)\left[\Theta(\kappa-k)\tilde{p}_{SS} - \Theta(k-\kappa)p_{SS}\right] \\
\frac{1}{r_a}\frac{dl_{II}}{dt} &= \sum_k \phi\rho_I(k)\left[\Theta(\kappa-k)\tilde{p}_{II} - \Theta(k-\kappa)p_{II}\right].
\end{aligned} \tag{A6}$$

To simplify, we absorb the sums of k into simplified expressions:

$$\begin{aligned}
\Sigma_S^- &\equiv \sum_k \rho_S(k)\Theta(k-\kappa)(-1), \\
\Sigma_S^+ &\equiv \sum_k \rho_S(k)\Theta(\kappa-k)(+1), \\
\Sigma_S &\equiv \Sigma_S^+ + \Sigma_S^- = \sum_k \rho_S(k)\mathrm{sgn}(\kappa-k), \\
\Sigma_I &\equiv \sum_k \rho_I(k)\mathrm{sgn}(\kappa-k).
\end{aligned} \tag{A7}$$

To continue, we approximate the local degrees of the susceptibles by the global averages. For the susceptibles we replace $(k_S, k_I) \to (l_{SS}, l_{SI})$ so that the probabilities in Eq. 7, 8 of main text become independent of k

$$p_{SI} = 1 - p_{SS} \to \frac{\gamma l_{SI}}{\gamma l_{SI} + l_{SS}}; \quad \tilde{p}_{SS} = 1 - \tilde{p}_{SI} \to \frac{\gamma l_{SS}}{\gamma l_{SS} + l_{SI}}. \tag{A8}$$

Similarly, for the infected, we use $k_S \to l_{IS}(= l_{SI}),\ k_I \to l_{II}$ so that

$$p_{IS} = 1 - p_{II} = \tilde{p}_{IS} = 1 - \tilde{p}_{II} \to \frac{l_{SI}}{l_{SI} + l_{II}} \tag{A9}$$

Since the degree distributions also vary with time, the sums in Eq. A7 cannot be expressed in terms of the mean field variables $(\phi, l_{SI}, l_{SS}, l_{II})$ on which we have chosen to focus. To proceed, we make a further (drastic) assumption, that each ρ can be approximated by the Laplacian distribution of Section II.A, around the instantaneous $\kappa\,(\phi)$. Technically, this assumption gives rise to an unphysical constraint, namely, *symmetric* degree distributions conserves the total number of links. But, this contradicts Eqn. (A3), since $\kappa(\phi(t))$ cannot be a constant. To ensure that, during adaptations, $l_{SI} + l_{SS} + l_{II} = \kappa(\phi)/2$ is satisfied, we introduce an auxiliary 'damping' field:

$$\eta = -r_a(l_{SI} + l_{SS} + l_{II} - \kappa(\phi)/2) \tag{A10}$$

into the evolution equations of the l's. With these modifications, the final set of mean field equations for

the links (including recovery, infection and network adaptation process) read:

$$
\begin{aligned}
\frac{dl_{SI}}{dt} &= \alpha\eta + r_a \left[(1-\phi)\left(\tilde{p}_{SI}\Sigma_S^+ + p_{SI}\Sigma_S^-\right) + \phi p_{IS}\Sigma_I\right] - \mu l_{SI} + 2\mu l_{II} + 2\lambda\frac{l_{SS}l_{SI}}{1-\phi} - \lambda\frac{l_{SI}^2}{1-\phi} - \lambda l_{SI} \\
\frac{dl_{SS}}{dt} &= (1-\alpha-\beta)\eta + r_a(1-\phi)\left(\tilde{p}_{SS}\Sigma_S^+ + p_{SS}\Sigma_S^-\right) + \mu l_{SI} - 2\lambda\frac{l_{SS}l_{SI}}{1-\phi} \\
\frac{dl_{II}}{dt} &= \beta\eta + r_a\phi p_{II}\Sigma_I - 2\mu l_{II} + \lambda\frac{l_{SI}^2}{1-\phi} + \lambda l_{SI},
\end{aligned}
\tag{A11}
$$

where the 'damping coefficients' α, β are somewhat arbitrary. They must be chosen to model the fact that, as the infection rages, SI and II links should decrease while SS links should increase. Thus, we impose $\alpha, \beta > 0$ and $\alpha + \beta > 1$. The link equations in A11 along with the node Eqn.A4 and fear function Eq. 5 in main text forms the set of mean field equations for selective adaptations.

We evolve the mean field equations numerically and obtain the stationary state infection and links. The time of evolution varied from 500-1000 units for reaching steady state. We chose $\alpha = 1.0$, $\beta = 0.5$, $\lambda/\mu \in [0, 2]$ and a range of initial infections so as to find the various stable fixed points shown in the text. The remaining parameters are the same as those used for Monte-Carlo simulations.

References

1. Zanette D, Risau-Gusmán S (2008) Infection spreading in a population with evolving contacts. Journal of biological physics 34: 135–148.

2. Gross T, D'Lima CJD, Blasius B (2006) Epidemic dynamics on an adaptive network. Phys Rev Lett 96: 208701.

3. Keeling M, Eames K (2005) Networks and epidemic models. Journal of the Royal Society Interface 2: 295–307.
